# Robust Expression of Functional NMDA Receptors in Human Induced Pluripotent Stem Cell-Derived Neuronal Cultures Using an Accelerated Protocol

**DOI:** 10.3389/fnmol.2021.777049

**Published:** 2021-11-26

**Authors:** Jacob B. Ruden, Mrinalini Dixit, José C. Zepeda, Brad A. Grueter, Laura L. Dugan

**Affiliations:** ^1^Vanderbilt Brain Institute, Vanderbilt University, Nashville, TN, United States; ^2^Division of Geriatric Medicine, Department of Medicine, Vanderbilt University Medical Center, Nashville, TN, United States; ^3^Department of Pharmacology, Vanderbilt University, Nashville, TN, United States; ^4^Department of Anesthesiology, Vanderbilt University Medical Center, Nashville, TN, United States; ^5^Department of Psychiatry, Vanderbilt University Medical Center, Nashville, TN, United States; ^6^Vanderbilt Center for Addiction Research, Vanderbilt University, Nashville, TN, United States; ^7^Department of Molecular Physiology and Biophysics, Vanderbilt University, Nashville, TN, United States; ^8^VA Tennessee Valley Geriatric Research, Education, and Clinical Center (GRECC), Nashville, TN, United States

**Keywords:** calcium flux, induced pluripotent stem cells (iPSCs), neural progenitor cells (NPCs), neurons, NMDA receptors

## Abstract

N-methyl-D-aspartate (NMDA) receptors are critical for higher-order nervous system function, but in previously published protocols to convert human induced pluripotent stem cells (iPSCs) to mature neurons, functional NMDA receptors (NMDARs) are often either not reported or take an extended time to develop. Here, we describe a protocol to convert human iPSC-derived neural progenitor cells (NPCs) to mature neurons in only 37 days. We demonstrate that the mature neurons express functional NMDARs exhibiting ligand-activated calcium flux, and we document the presence of NMDAR-mediated electrically evoked postsynaptic current. In addition to being more rapid than previous procedures, our protocol is straightforward, does not produce organoids which are difficult to image, and does not involve co-culture with rodent astrocytes. This could enhance our ability to study primate/human-specific aspects of NMDAR function and signaling in health and disease.

## Introduction

*N*-methyl-D-aspartate (NMDA) receptors are ionotropic glutamatergic receptors which are critical for neurotransmission and higher-order function of the nervous system, including long-term potentiation (LTP), memory formation and consolidation, and maintenance of neuronal plasticity (Liu et al., 2007; Hunt and Castillo, 2012; Luscher and Malenka, 2012; Paoletti et al., 2013; Chakraborty et al., 2017). Activation of NMDA receptors (NMDARs) requires both the receptor ligand, glutamate, and a depolarizing stimulus to release Mg^2+^ inhibition of the receptor, and thus are often referred to as “coincidence detectors” (Hunt and Castillo, 2012; Luscher and Malenka, 2012). One defining hallmark of NMDAR activation is flux of Ca^2+^ through the ion channel to produce local increases in intracellular Ca^2+^, and subsequent activation of calcium-dependent signaling pathways (Liu et al., 2007; Hunt and Castillo, 2012; Luscher and Malenka, 2012).

Much of our understanding of NMDAR function has come from studies on cell cultures from rodents and other non-primate animal model systems, or from immortalized cell lines with induced expression of human NMDAR subunits. However, emerging evidence suggests that there are important genetic, molecular, and functional differences between NMDARs in primates, including humans, and other species, specifically rodents, which may modify NMDAR composition, activation, and downstream signaling. For example, differences between mice and humans in protein abundance of postsynaptic density factors found in the NMDAR complex have been reported (Bayes et al., 2012). These differences could have major implications for understanding the roles of NMDARs in health and disease and as drug targets. Thus, the ability to study human NMDARs in the context of all the regulatory and co-activating factors necessary for human neuronal function may be critical to understanding the roles of NMDARs in physiologic and pathophysiologic conditions.

Human neurons grown from induced pluripotent stem cells (iPSCs) provide an excellent opportunity to study human NMDARs in health and disease. However, only a limited number of published protocols have documented maturation of NMDARs molecularly or reported Ca^2+^ influx attributed to functional NMDARs; these protocols have limitations, including culturing cells for long periods of time (Lieberman et al., 2012; Zhang et al., 2016; Ishii et al., 2017; Pruunsild et al., 2017), co-culturing with rodent astrocytes (Shcheglovitov et al., 2013; Lam et al., 2017), or culturing 3-D organoids (Yakoub and Sadek, 2018, 2019; Zafeiriou et al., 2020; Gordon et al., 2021) which are challenging to image (Rios and Clevers, 2018; Booij et al., 2019; Kassis et al., 2019).

Additionally, the glutamate-dependent currents that were identified *via* electrophysiology in many of the protocols cited above could have been evoked by either sodium or calcium. Sodium influx through NMDARs is likely dramatically greater than calcium influx, as NMDAR activation produces intracellular sodium increases in the millimolar range and intracellular calcium increases in the nanomolar range (Xin et al., 2005). In contrast, calcium imaging exclusively identifies calcium flux through NMDARs.

Our interest in NMDARs in nervous system disease led us to develop an accelerated protocol to produce robust monolayer cultures with functional human NMDARs. The cultures demonstrate increased intracellular Ca^2+^ in response to NMDA and exhibit NMDAR-mediated electrically evoked postsynaptic current. Our procedure starts with iPSC-derived neural progenitor cells (NPCs; defined as SOX1- and Nestin-positive cells). We successfully converted 3 separate iPSC-derived NPC lines to mature, fully functional neurons with our protocol.

## Methods

### Reagents and Antibodies

6-well cell culture plates, Matrigel, and Laminin were purchased from Corning. Poly-L-ornithine hydrobromide (PLO), cAMP, L-Ascorbic Acid, paraformaldehyde, dimethyl sulfoxide, and RIPA Buffer were purchased from Sigma. DMEM/F12, B-27 Supplement, and GlutaMAX Supplement were purchased from Gibco. 35 mm glass bottom dishes were purchased from MatTek. TRIzol Reagent and Fluo-4, AM were purchased from Invitrogen. OneStep reverse transcriptase-polymerase chain reaction (RT-PCR) Kit was purchased from Qiagen. Primers were purchased from Integrated DNA Technologies. cOmplete, Mini Protease Inhibitor Cocktail was purchased from Roche. Pierce BCA Protein Assay Kit and SuperSignal West Femto Maximum Sensitivity Substrate were purchased from Thermo Fisher Scientific.

NMDAR1, NMDAR2A, MAP2, and GFAP antibodies were purchased from abcam (Cambridge, MA, United States). TUJ1 antibody was purchased from Neuromics (Edina, MN, United States). PSD95 antibody was purchased from Cell Signaling Technology (Danvers, MA, United States). Synaptotagmin-1 antibody was purchased from Developmental Studies Hybridoma Bank (Iowa City, IA, United States). NMDAR2B antibody was purchased from BD Transduction Laboratories (San Jose, CA, United States). GFAP antibody was purchased from Calbiochem (Burlington, MA, United States). All fluorescent secondary antibodies were purchased from Life Technologies (Waltham, MA, United States). Mouse and rat HRP secondary antibodies were purchased from Invitrogen (Waltham, MA, United States).

### Cell Culture

A cryopreserved human NPC line derived from female human iPSCs (XCL-4; STEMCELL Technologies, Vancouver, 70902) was obtained. These NPCs are provided as greater than or equal to 90% SOX1-positive and Nestin-positive cells. The XCL-4 line was expanded in Neural Progenitor Medium 2 (STEMCELL Technologies, 08560) or in NSC Maintenance Medium with Supplements A and B (XCell Science, Novato, CA, United States, SM-001-BM100, SM-001-SA100, and SM-001-SB100) on 6-well cell culture plates (Corning, 353846) coated with Matrigel (Corning, 354277). Neural Progenitor Medium 2 was discontinued, which necessitated the media switch. After three passages, cells were plated on 6-well plates coated with 15 μg/mL PLO (Sigma, P3655) and 10 μg/mL Laminin (Corning, 354232) at a plating density of 5 × 10^4^ cells per cm^2^. Cells were fed every day with STEMdiff Neuron Differentiation medium (STEMCELL Technologies, 08500) or STEMdiff Forebrain Neuron Differentiation medium (STEMCELL Technologies, 08600) for 6–7 days until cells were 90–95% confluent. STEMdiff Neuron Differentiation medium was discontinued, which necessitated the media switch. Cells were treated with ACCUTASE Cell detachment solution (STEMCELL Technologies, 07920) for 5–10 min, washed with DMEM/F12 (Gibco, 11330-032) and pelleted by centrifugation at 1,500 × *g* for 5 min at room temperature. Cells were resuspended in STEMdiff Neuron Maturation medium (STEMCELL Technologies, 08510) or STEMdiff Forebrain Neuron Maturation medium (STEMCELL Technologies, 08605) and were plated onto either 35 mm glass bottom dishes (for confocal imaging; MatTek, P35G-1.5-14-C) or 6-well plates (for Western blot and RT-PCR) coated with PLO/Laminin at a plating density of 2.5–5 × 10^5^ cells per dish or well. STEMdiff Neuron Maturation medium was discontinued, which necessitated the media switch.

In one experiment, cells were fed every other day with STEMdiff Neuron Maturation medium for the entire maturation period. For all other experiments, cells were fed every other day with STEMdiff Neuron Maturation medium or STEMdiff Forebrain Neuron Maturation medium for 7 days and then were fed every other day with Neurobasal Medium (Gibco, 21103-049) or (for cultures after STEMdiff Neuron Differentiation medium and STEMdiff Neuron Maturation medium were discontinued) BrainPhys Neuronal Medium (STEMCELL Technologies, 05790) supplemented with B-27 Supplement (Gibco, 17504-044) or NeuroCult SM1 Neuronal Supplement (STEMCELL Technologies, 05711), GlutaMAX Supplement (Gibco, 35050-061), 20 ng/mL BDNF (STEMCELL Technologies, 78005), 0.5 mM cAMP (Sigma, D0627), 0.2 mM L-Ascorbic acid (Sigma, A8960), and 10 μg/mL Laminin. Cells were in maturation media for 25–45 days.

### Reverse Transcriptase-Polymerase Chain Reaction

Total RNA was isolated by adding TRIzol Reagent (Invitrogen, 15596026) to each well, and RNA extraction was performed according to the manufacturer’s protocol. Total RNA concentration was measured using a NanoDrop. 0.5 μg of RNA was used to generate cDNA, and the QIAGEN OneStep RT-PCR Kit (210212) was used to amplify the cDNA. A final volume of 25 μL per PCR reaction was used. We used the GluN1, GluN2A, GluN2B, GluN2C, GluN2D, GluN3A, and GluN3B human target cDNA primers listed in Lee et al. (2010), and, as a control, the GAPDH primer listed in Behrens et al. (2008) to measure RNA expression. The final product was detected using a 2% agarose gel with ethidium bromide.

### Western Blot

Cells were rinsed twice with PBS and were scraped in PBS before being pelleted by refrigerated centrifugation. The supernatant was discarded, and cell pellets were resuspended in RIPA Buffer (Sigma, R2078) containing 1% SDS and cOmplete, Mini Protease Inhibitor Cocktail (Roche, 4693124001). Cells were lysed by sonication on ice.^1^ Note that supernatant and particulate were not separated. Protein concentration was measured using the Pierce BCA Protein Assay Kit (Thermo Fisher Scientific, 23225). Total cell lysates were stored at −80°C. 10% SDS-PAGE gels were used. Gels were transferred overnight at 4°C onto PVDF membranes. Membranes were blocked in 5% milk in PBST for 1 h and incubated overnight with NMDAR1 (abcam, ab109182, RRID:AB_10862307, 1:1000 dilution), NMDAR2A (abcam, ab124913, RRID:AB_10975154, 1:1000 dilution), NMDAR2B (BD Transduction Laboratories, 610417, RRID:AB_397797, 1:500 dilution), and GAPDH (Invitrogen, MA5-15738, RRID:AB_10977387, 1:5000 dilution) primary antibodies in 5% milk in PBST. Membranes were washed three times with PBST, incubated in corresponding HRP secondary antibodies in PBST for 1 h at room temperature, and washed three times with PBST before visualizing using SuperSignal West Femto Maximum Sensitivity Substrate (Thermo Fisher Scientific, 34095) according to the manufacturer’s protocol using a G:BOX and the associated GeneSys software (Syngene, RRID:SCR_015770).

### Immunohistochemistry

Cells cultured on 35 mm glass bottom dishes were fixed with 4% paraformaldehyde (Sigma, 158127) for 15–30 min and then washed 2–3 times with PBS. Cells were permeabilized with 2% BSA and 0.3% Triton X-100 for 30 min. Cells were washed twice with PBS containing 2% BSA and were incubated with TUJ1 (Neuromics, CH23005, RRID:AB_2210684, 1:2000 dilution; or BioLegend, 801202, RRID:AB_10063408, 1:1000 dilution), MAP2 (abcam; ab32454, RRID:AB_776174, 1:2000 dilution), NMDAR1 (BioLegend, 818601, RRID:AB_2564822, 1:400 dilution), NMDAR2A (Millipore, AB1555P, RRID:AB_90770, 1:200 dilution), NMDAR2B (Millipore, AB1557P, RRID:AB_90772, 1:200 dilution), PSD95 (Cell Signaling Technology, 3450, RRID:AB_2292883, 1:200 dilution), synaptotagmin-1 (Developmental Studies Hybridoma Bank, mAB 30, RRID:AB_2295002, 1:200 dilution), and/or GFAP (Calbiochem, 345860, RRID:AB_2109651, 1:2000 dilution) primary antibodies in PBS containing 2% BSA overnight. Cells were washed twice with PBS containing 2% BSA, were incubated with corresponding fluorescent secondary antibodies for 1 h at room temperature, and then washed three times with PBS and treated with DAPI before imaging on an LSM 880/Axio Observer.Z1 confocal microscope (Zeiss). Images were processed in Fiji (RRID:SCR_002285).

### Calcium Imaging

One vial of Fluo-4, AM (Invitrogen, F14201) was dissolved in 50 μL of dimethyl sulfoxide (Sigma, D2650) before being added to 10 mL of HEPES-bicarbonate balanced salt solution with 5.5 mM D-glucose (HBBSS_5_._5_) (Giffard et al., 1992). Cells cultured on 35 mm glass bottom dishes were incubated with Fluo-4, AM in HBBSS_5_._5_ for 30 min in the CO_2_ incubator before being washed once with 1 mL of HBBSS_5_._5_. When ready to image on an LSM 880/Axio Observer.Z1 confocal microscope (Zeiss), 10 μL of 10 mM glycine (final concentration: 100 μM; RPI, G36050) was added to the dish for 5 min at room temperature. Time series imaging was performed with an interval of 1–3 min per image. Two baseline images were taken prior to the addition of 100 μL of vehicle (HBBSS_5_._5_) or 0.5–3 mM NMDA (final concentration: 50–300 μM; Sigma, M3262). In some cultures, 100 μL of 100 μM of the NMDAR antagonist MK-801 (final concentration: 10 μM; Sigma, M107) was added after additional images were taken.

### Calcium Imaging Analysis

For each calcium imaging experiment, using MetaMorph (Molecular Devices, RRID:SCR_002368), brightness was artificially increased and individual cells (identified by presence of Fluo-4) in the first image were circled. Brightness was then reset to normal and Fluo-4 fluorescence intensity was logged for every cell for each image of the experiment. Intensity values for the first two images of every cell were averaged, with this value being considered the baseline fluorescence intensity. Data was normalized to this baseline.

### Electrophysiology

Whole-cell voltage clamp recordings were performed on XCL-4 derived mature cultures (37–39 days in maturation media) transferred into artificial cerebral spinal fluid (in mM: 119 NaCl, 2.5 KCl, 1.3 MgCl_2_-6H_2_O, 2.5 CaCl_2_-2H_2_O, 1.0 NaH_2_PO_4_-H_2_O, 26.2 NaHCO_3_, and 11 glucose; 290–295 mOsm) and patched with 4–6 MΩ recording pipettes (pulled with a P-1000 Micropipette Puller; Sutter Instrument) using a Cs^+^-based intracellular solution (in mM: 120 CsMeSO_3_, 15 CsCl, 8 NaCl, 10 HEPES, 0.2 EGTA, 10 TEA-Cl, 4.0 Mg-ATP, 0.3 Na-GTP, 0.1 spermine, and 5.0 QX 314 bromide; 290 mOsm). NMDAR-mediated electrically evoked excitatory postsynaptic currents were obtained at + 40 mV using a parallel bipolar electrode (FHC Worldwide, customized 30210-PBSA1045) and isolated with the GABA_A_ receptor antagonist, picrotoxin (final concentration: 50 μM; Sigma), and the AMPA receptor antagonist, NBQX (final concentration: 5 μM; Tocris Bioscience), and were then blocked using the NMDAR antagonist, APV (final concentration: 50 μM; Tocris Bioscience). Inclusion criteria for cells were a steady (<20% change) access resistance (R_A_) of <16 mΩs, and a steady holding current of <−100 pA at −70 mV.

### Statistical Analysis

All statistics were performed using SigmaPlot 14.5 (Inpixon, Palo Alto, CA, United States, RRID:SCR_003210). One-way analysis of variance (ANOVA) and Holm-Sidak tests were performed for Figure 3C. One-way repeated measures ANOVA and Bonferroni tests were performed for Figure 4D. Significant differences were noted if *p* < 0.05.

**FIGURE 1:**
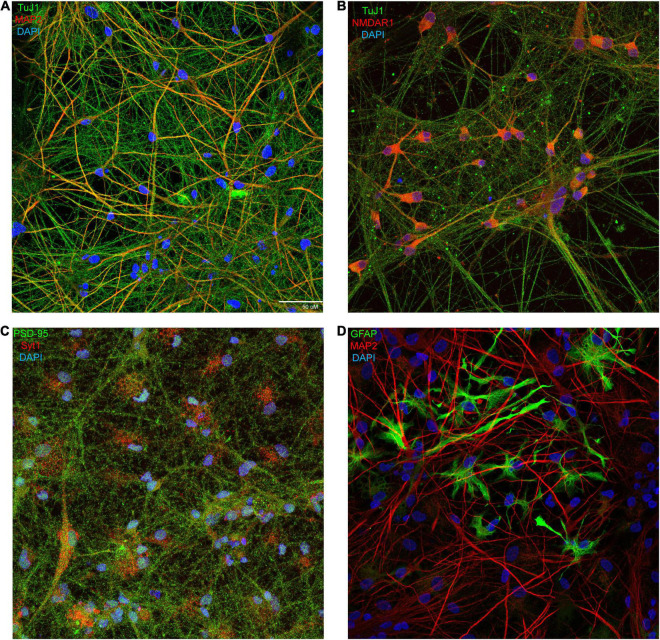
Immunofluorescent confocal imaging and characterization of mature human neuronal cultures. **(A–D)** XCL-4 derived mature cultures (28–37 days in maturation media) were fixed, permeabilized, and stained for the mature excitatory neuron markers β-tubulin III [TuJ1; green; **(A,B)**] and microtubule-associated protein 2 [MAP2; red; **(A,D)**], the obligate NMDAR subunit, NMDAR1 [red; **(B)**], the presynaptic marker synaptotagmin 1 [Syt1; red; **(C)**], the postsynaptic marker postsynaptic density protein 95 [PSD-95; green; **(C)**], and the astrocytic marker glial fibrillary acidic protein [GFAP; green; **(D)**]. All cultures were counterstained with the nuclear stain DAPI [blue; **(A–D)**].

**FIGURE 2:**
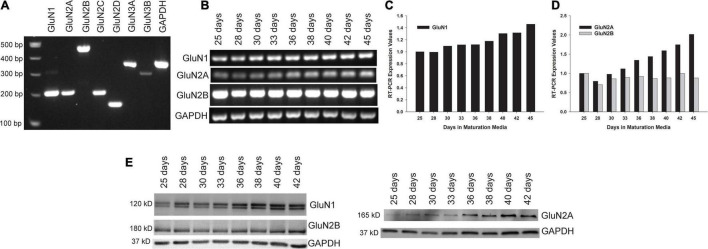
Expression of NMDAR subunits in maturing human neuronal cultures. **(A)** Reverse transcriptase-polymerase chain reaction (RT-PCR) was performed with RNA harvested from XCL-4 derived mature neuronal cultures (36 days in maturation media) to confirm the expression of several of the NMDAR subunits. **(B–D)** A time course study was performed with RNA harvested from cultures in maturation media at increasing times starting at 25 days to assess NMDAR subunit expression **(B)** and displayed quantitatively **(C,D)**. **(E)** NMDAR subunit protein expression was assessed by Western immunoblot at various stages of culture maturation.

**FIGURE 3:**
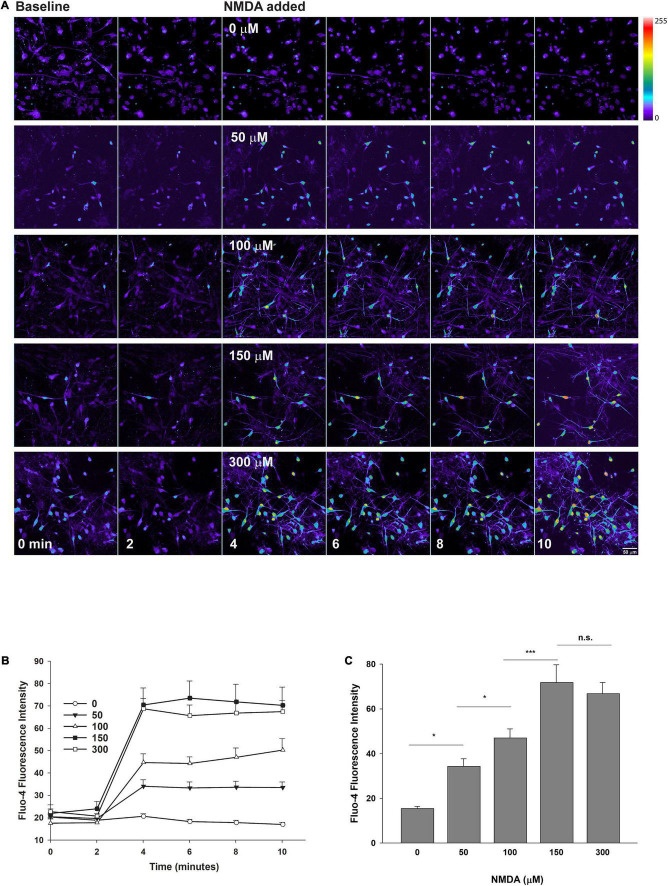
Confocal imaging of intracellular calcium in human neurons exposed to NMDA. XCL-4 derived mature cultures (41–42 days in maturation media) were loaded with the calcium indicator Fluo-4 for 30 min and then washed with HBBSS_5_._5_ media, followed by the addition of glycine (final concentration of 100 μM) prior to imaging. Using a time series protocol at 40× magnification, images were taken every 2 min. Two baseline images were taken prior to the addition of vehicle (HBBSS_5_._5_), or increasing final concentrations of NMDA by bath application, and then four additional images were taken. **(A)** NMDA dose-response montages are shown. Images were converted to a linear pseudocolor scale using MetaMorph. Individual cells were identified and Fluo-4 fluorescence intensity at each time point was measured. The intensity of cells at each time point were averaged together. **(B,C)** Data were graphed using SigmaPlot 12 [full time series from one experiment **(B)**; 10-min time point from five independent replicates **(C)**] and one-way analysis of variance (ANOVA) and Holm-Sidak tests were performed for panel **(C)** using SigmaPlot 14.5. Data are represented as mean + SEM. ***P < 0.001; *P < 0.05; n.s., not significant. *n* = 32–74 cells per condition.

**FIGURE 4:**
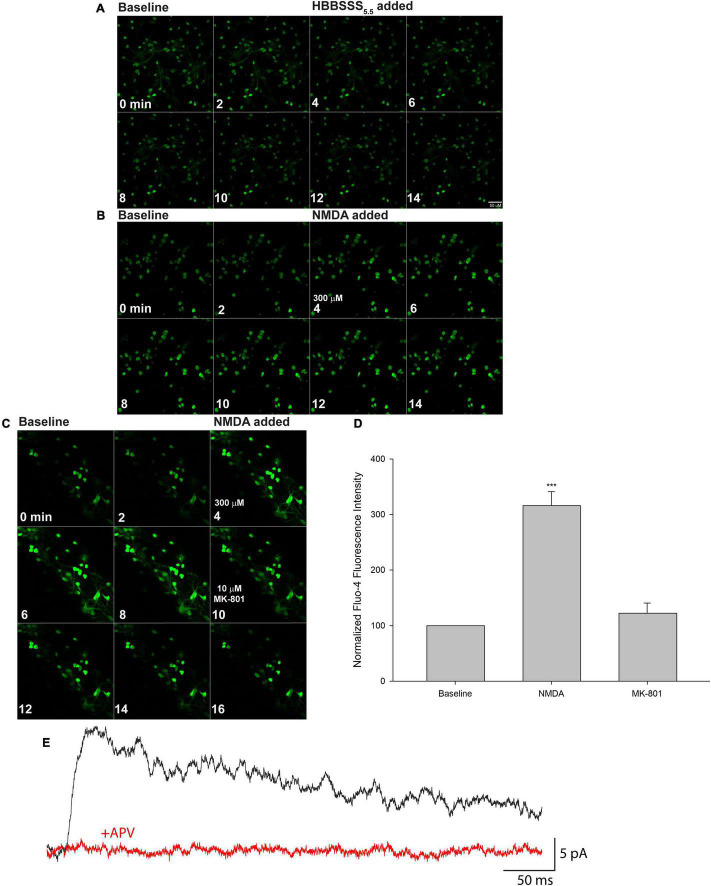
Mature iPSC-derived neuronal cultures express functional NMDA receptors (NMDARs). XCL-4 derived mature cultures (30–39 days in maturation media) were loaded with the calcium indicator Fluo-4 for 30 min and then washed with HBBSS_5_._5_ media, followed by the addition of glycine (final concentration of 100 μM) prior to imaging. Using a time series protocol at 40× magnification, two baseline images were taken prior to the addition of vehicle (HBBSS_5_._5_), or a final concentration of 300 μM NMDA. For some cultures, the NMDAR antagonist MK-801 at a final concentration of 10 μM was added after additional images were taken. **(A–C)** Representative montages are shown. Fluo-4 time series images were analyzed using MetaMorph. Individual cells were identified and Fluo-4 fluorescence intensity at each time point was measured. The intensity of cells at each time point were averaged together. The first two time points were averaged as the baseline intensity, and all data were normalized to the corresponding baseline. **(D)** Data from the montage shown in panel **(C)** were graphed using SigmaPlot 14.5. One-way repeated measures ANOVA and Bonferroni tests were performed using SigmaPlot 14.5. Data are represented as mean + SEM. ***P < 0.001. *n* = 21 cells per condition. **(E)** A representative trace is shown of an NMDAR-mediated electrically evoked postsynaptic current from XCL-4 derived mature cells in the presence of the GABA_A_ receptor antagonist, picrotoxin (final concentration: 50 μM), and the AMPA receptor antagonist, NBQX (final concentration: 5 μM), before (black) and after (red) application of the NMDAR antagonist, APV (final concentration: 50 μM).

## Results

### Development and Maturation of Induced Pluripotent Stem Cell-Derived Neuronal Cultures

We chose to establish a protocol which utilized NPCs derived from human iPSCs (Supplementary Figure 1). Three lines were used (Supplementary Figures 2, 3). Immunohistochemistry of XCL-4 converted mature cell cultures exhibited the presence of β-tubulin III (Figures 1A,B), microtubule-associated protein 2 (Figures 1A,D), NMDAR1 (Figure 1B and Supplementary Figure 4), NMDAR2A (Supplementary Figure 4), NMDAR2B (Supplementary Figure 4), synaptotagmin 1 (Figure 1C), and postsynaptic density protein 95 (Figure 1C), which are markers indicative of mature neurons. GFAP-positive astrocytes (Figure 1D) are also present in the same converted cultures. We observed that the cultures contain approximately 20–30% astrocytes (based on counts of DAPI and GFAP from four different fields of the same dish).

### Developmental Expression of Markers of Mature NMDA Receptors

Mature XCL-4 derived neurons were harvested at various maturation days to determine NMDAR subunit expression by RT-PCR (Figures 2A–D) and by Western blot (Figure 2E).

### Presence of Functional NMDA Receptors in the Mature Cultures

To confirm that the NMDARs in the XCL-4 converted cultures are functional, we performed Fluo-4 fluorescent calcium imaging and found that NMDA treatment enhances calcium signaling in the cultures (Figures 3A–C, 4B,D), while treatment with an NMDAR antagonist inhibits calcium signaling (Figures 4C,D). Vehicle treatment has no effect on calcium signaling (Figures 3A–C, 4A). To validate that the NMDARs in our cultures are functional at the synapse, we performed whole-cell voltage clamp electrophysiology. In the presence of GABA_A_ receptor and AMPA receptor antagonists, electrical stimulation elicited a postsynaptic current (mean peak = 16.99 pA, SD = 8.96 pA, *n* = 7 cells) sensitive to an NMDAR antagonist (Figure 4E).

## Discussion

Experiments utilizing neurons derived from human iPSCs are critical to ultimately treat and prevent human disease, as there are many important differences between human and rodent neurons, including regarding their NMDARs. For example, a primate-specific short isoform of the NMDAR 2A subunit (GluN2A-S) was recently identified which can co-assemble to form a functional NMDAR, but whose function has not yet been defined (Warming et al., 2019). Additionally, while the GluN2B to GluN2A developmental switch is evolutionarily conserved (Paoletti et al., 2013), this switch occurs earlier in humans than in rodents (Bar-Shira et al., 2015). Also, GluN2A and GluN2C subunits are present before birth in humans but not in rodents (Watanabe et al., 1992; Haberny et al., 2002). Sequence identity between rat and human GluN2C subunits is only 87.1%, and the sequence identity of the carboxy-terminal domains between rat and human GluN2C subunits is only 71%, suggesting that differences in membrane trafficking and phosphorylation may exist (Hedegaard et al., 2012). Two GluN2C/D selective modulators were found to be less potent for rat GluN2C-containing receptors compared to human GluN2C-containing receptors (Hedegaard et al., 2012).

Our protocol yields forebrain-type neurons, like other protocols (Muratore et al., 2014; Zhang et al., 2018; Bell et al., 2019). The vast majority of neurons are excitatory. Our protocol generates mature neurons in only 37 days (approximately 5 weeks), while other protocols culture cells for 7–12 weeks or longer before mature neurons are generated (Lieberman et al., 2012; Zhang et al., 2016; Ishii et al., 2017; Pruunsild et al., 2017). Additionally, our XCL-4 converted cultures contain both neurons and astrocytes derived from the same human cell line. Astrocytes are an important nervous system component, as they are closely associated with and can alter the function of synapses (Chung et al., 2015; Farhy-Tselnicker and Allen, 2018). The absence of astrocytes from other species in our cultures, unlike other protocols that co-culture with rodent astrocytes (Shcheglovitov et al., 2013; Lam et al., 2017), is noteworthy, as there are other key differences between human and animal model systems that are crucial to understand age-related effects on brain. Inflammation and activation of innate immunity are believed to underlie much of aging biology. However, humans express several inflammatory mediators that are not present in rodents. For example, NADPH oxidase 5 (NOX5), which is calcium-dependent, is expressed in primates, but not rodents or lower organisms (Touyz et al., 2019). NOX5’s expression in human neurons, and responsiveness to calcium to induce free radical production, could contribute importantly to inflammation in the brain, yet is most likely to be studied effectively only in human (or other primate) cells. Thus, there is a pressing need for models which retain the features unique to humans to allow for accurate characterization of human NMDARs in both physiologic and pathophysiologic conditions.

Our protocol begins with cell lines at the NPC stage. The rationale for starting with NPCs is several-fold. There are multiple published procedures for conversion of iPSCs to NPCs (Mertens et al., 2016). Working with NPCs as the starting lineage allows quick expansion of the NPC line from frozen aliquots, and, importantly, many institutions have turned to core facilities to perform the initial collection of patient tissue, conversion to iPSCs, and differentiation to tissue-specific precursor stem cell lines, with these cell lines provided to the end-user. This is because of the increased patient protection and consenting requirements for iPSC generation and specialized requirements for iPSC viral transformation that are difficult for individual labs to provide (Lowenthal et al., 2012; Hu, 2014; Santostefano et al., 2015).

We utilized calcium imaging and electrophysiology to confirm that the NMDARs in our cultures were functional. Studying calcium dynamics on the order of minutes allows enough time for the addition of multiple substrates (such as NMDA and then MK-801) to the neuronal cultures being imaged. Studying slower calcium dynamics also does not require the elaborate and expensive setups used by those who study fast calcium dynamics. Even though we found the expression of various NMDAR subunits at maturation day 28, calcium imaging performed at that time point did not show the presence of robust functional NMDARs (data not shown). Characterization of cultures derived from a new protocol must include functional assays such as calcium imaging and electrophysiology to fully assess the conditions of the cultures.

We also converted patient fibroblast (BV3525A#1 iPSC line) derived NPCs to neurons with functional NMDARs in a short period of time. Our current protocol provides a basis for future drug targeting and screening for neurological diseases in a considerably shorter time frame. Soon, we envision that a patient presenting with a disease where impaired NMDARs may be implicated can have their tissue samples collected. Patient-derived iPSCs will immediately be generated and converted into mature neurons within weeks for specific therapeutic testing. The results of this testing will impact the future treatment plan of that same patient. In the same way that many cancer treatments are now personalized, we hope that personalized treatments for neurological disorders can be similarly achieved.

## Data Availability Statement

The raw data supporting the conclusions of this article will be made available by the authors, without undue reservation.

## Author Contributions

MD, JR, JZ, BG, and LD designed the experiments and interpreted the results. MD, JR, and JZ conducted the experiments. JR, MD, JZ, and LD wrote the manuscript. All authors contributed to the article and approved the submitted version.

## Conflict of Interest

The authors declare that the research was conducted in the absence of any commercial or financial relationships that could be construed as a potential conflict of interest.

## Publisher’s Note

All claims expressed in this article are solely those of the authors and do not necessarily represent those of their affiliated organizations, or those of the publisher, the editors and the reviewers. Any product that may be evaluated in this article, or claim that may be made by its manufacturer, is not guaranteed or endorsed by the publisher.

## Footnotes

1
https://www.abcam.com/ps/products/133/ab133625/documents/Lysate%20Preparation%20Protocol%20v3%20(website).pdf
