# Supplementary material for: Robust Expression of Functional NMDA Receptors in Human Induced Pluripotent Stem Cell-Derived Neuronal Cultures Using an Accelerated Protocol
Source: Front Mol Neurosci. 2021 Nov 26;14:777049. doi: 10.3389/fnmol.2021.777049 (PMC8661903; doi:10.3389/fnmol.2021.777049)
Supplement: Supplementary file 1 [file Data_Sheet_1.DOCX]

Supplementary Material

# List of Supplementary Figures

Supplementary Figure 1. Protocol sequence for differentiation and maturation of human neuronal cultures.

Supplementary Figure 2. Immunofluorescent confocal imaging of mature iPSC-derived neuronal cultures.

Supplementary Figure 3. Mature patient iPSC-derived neuronal cultures express functional NMDA receptors.

Supplementary Figure 4. Immunofluorescent confocal imaging of NMDA receptor subunits in mature human neuronal cultures.

# Supplementary Figures

#

# Supplementary Figure 1. Protocol sequence for differentiation and maturation of human neuronal cultures. Cryopreserved vials of NPC lines were maintained in liquid nitrogen until use. After thawing, cells were plated for differentiation. Cells were transitioned through each step as depicted in this figure and as described in detail in Method.


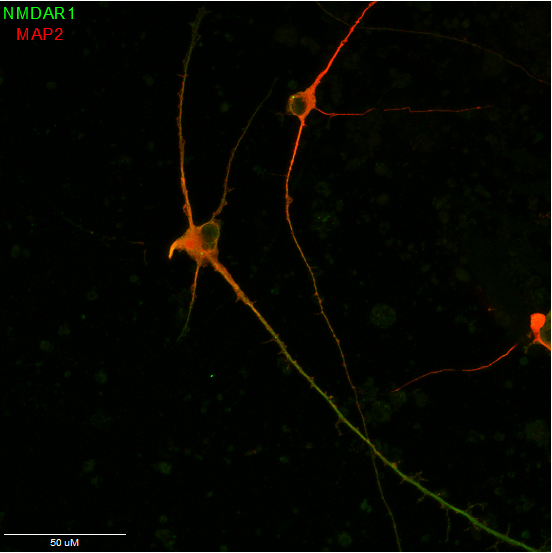


# Supplementary Figure 2. Immunofluorescent confocal imaging of mature iPSC-derived neuronal cultures. Using a well-established control female human iPSC line, CC3 (Kumar et al., 2014), and an earlier iteration of the protocol described in the main text, cells were converted into mature neurons and were fixed, permeabilized, and stained for the mature excitatory neuron markers MAP2 (red) and NMDAR1 (green). Cultures also exhibited extensive arborization of both axons and dendrites.

# Supplementary Figure 3. Mature patient iPSC-derived neuronal cultures express functional NMDA receptors. Mature cultures (37 days in maturation media) derived from the BV3525A#1 female human iPSC line were loaded with the calcium indicator Fluo-4 for 30 minutes and then washed with HBBSS_5.5_ media, followed by the addition of glycine (final concentration of 100 µM) prior to imaging. Using a time series protocol at 40X magnification, two baseline images were taken prior to the addition of a final concentration of 300 µM NMDA. The NMDAR antagonist MK-801 at a final concentration of 10 µM was added after additional images were taken. Images at baseline, after addition of NMDA, and after addition of MK-801, are shown.


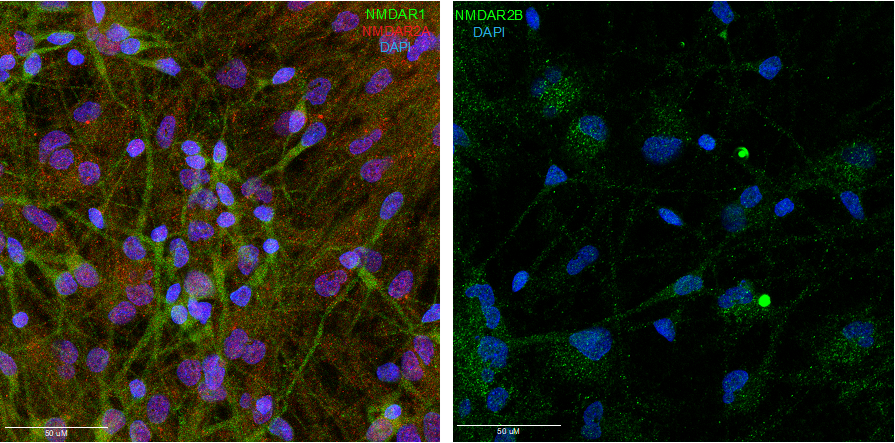


# Supplementary Figure 4. Immunofluorescent confocal imaging of NMDA receptor subunits in mature human neuronal cultures. XCL-4 derived mature cultures (37 days in maturation media) were fixed, permeabilized, and stained for NMDAR1 and NMDAR2A (green and red, respectively; left) and NMDAR2B (green; right), and counterstained with the nuclear stain DAPI (blue).

# Supplementary Reference

Kumar, K.K., Lowe, E.W., Jr., Aboud, A.A., Neely, M.D., Redha, R., Bauer, J.A., et al. (2014). Cellular manganese content is developmentally regulated in human dopaminergic neurons. *Sci Rep* 4**,** 6801. doi: 10.1038/srep06801.
